# Interventions to reduce the incidence of medical error and its financial burden in health care systems: A systematic review of systematic reviews

**DOI:** 10.3389/fmed.2022.875426

**Published:** 2022-07-27

**Authors:** Ehsan Ahsani-Estahbanati, Vladimir Sergeevich Gordeev, Leila Doshmangir

**Affiliations:** ^1^Department of Health Policy and Management, Tabriz Health Services Management Research Center, Iranian Center of Excellence in Health Management, School of Management and Medical Informatics, Tabriz University of Medical Sciences, Tabriz, Iran; ^2^Wolfson Institute of Population Health, Queen Mary University of London, London, United Kingdom; ^3^Department of Infectious Disease Epidemiology, London School of Hygiene and Tropical Medicine, London, United Kingdom; ^4^Social Determinants of Health Research Center, Tabriz University of Medical Sciences, Tabriz, Iran

**Keywords:** medical error, financial burden, hospital, intervention, quality of care, public health

## Abstract

**Background and aim:**

Improving health care quality and ensuring patient safety is impossible without addressing medical errors that adversely affect patient outcomes. Therefore, it is essential to correctly estimate the incidence rates and implement the most appropriate solutions to control and reduce medical errors. We identified such interventions.

**Methods:**

We conducted a systematic review of systematic reviews by searching four databases (PubMed, Scopus, Ovid Medline, and Embase) until January 2021 to elicit interventions that have the potential to decrease medical errors. Two reviewers independently conducted data extraction and analyses.

**Results:**

Seventysix systematic review papers were included in the study. We identified eight types of interventions based on medical error type classification: overall medical error, medication error, diagnostic error, patients fall, healthcare-associated infections, transfusion and testing errors, surgical error, and patient suicide. Most studies focused on medication error (66%) and were conducted in hospital settings (74%).

**Conclusions:**

Despite a plethora of suggested interventions, patient safety has not significantly improved. Therefore, policymakers need to focus more on the implementation considerations of selected interventions.

## Introduction

A medical error is a preventable adverse effect of medical care (“iatrogenesis”). It can be defined as the “failure of a planned action to be completed as intended or the use of a wrong plan to achieve an aim” (1). As the associated burden is evident, medical errors have drawn considerable attention from academics, hospital managers, and major health stakeholders. Medical errors have a significant adverse impact on patients' outcomes and workers' mental health. They are associated with a considerable financial burden and undermine public trust in the health system (2–4). Medical errors, including healthcare-related adverse events, occur in 8–12% of hospitalisations in Europe (5). At least 50% of hospitalized patients' harm could be preventable (6). Overall, healthcare-associated infections incidence is estimated at 4.1 million patients a year in Europe, with the four main types of error being urinary tract infections (27%), lower respiratory tract infections (24%), surgical site infections (17%), and bloodstream infections (10.5%) (5). In the US (2007), 1.7 million healthcare-associated infections occur annually. They result in excess healthcare costs of $35.7–$45 billion for inpatient hospital services (7, 8).

The medical errors can be classified based on their content or “what went wrong” (e.g., medication, surgical, transfusion, healthcare-associated infection) (9–15); location or “where did it happen” (e.g., intensive care unit, operation theater, emergency department, children's ward) (15–18); staff or “who made an error” (e.g., doctor, pharmacists, nurse) (10, 19, 20); error's severity or “how harmful was it” (e.g., error, no harm, near miss) (21–25); and “who was affected” (e.g., patient, family, medical staff) (26, 27). Depending on the type of medical errors, studies suggest various solutions, from simple activities (e.g., hand hygiene to prevent healthcare-associated infection) to more complex ones such as using technological instruments or methods to prevent retained surgical instruments errors (7, 15).

Despite the ongoing efforts to reduce and prevent the burden of medical errors and related patient harm, global efforts have not yet achieved substantial change over the past 15 years due to various reasons (6). Unclear policies, insufficient or unreliable data to drive patient safety improvements, unskilled health care professionals, lack of organizational leadership capacity, and non-participation of patients and families in the care process led to unsustainable and insignificant improvements in health care safety (2). Hence the primary goal of this article was to conduct a systematic review of systematic reviews to elicit interventions that can reduce medical errors or medical error costs in hospitals and analyse interventions implementation results where available. Specifically, we focused on interventions that can reduce health care costs, patient's harm and death, improve health services quality, patient's satisfaction, and safety.

## Methods

### Literature search and study selection

Inclusion criteria for articles considered in this review were as follows: (a) systematic reviews; (b) studies published in English language; (c) studies on solutions regarding medical error reduction or medical error costs; (d) studies on interventions in hospitals or the whole of the healthcare sector, which entered the study regardless of whether these reviews were based on reported errors, an examination of medical profiles, observational studies or other methods. We excluded studies (a) published in languages other than English; (b) studies conducted in settings other than the hospital; (c) studies focused only on a specific type of medical error/activity/patient subgroup, or a sporadic type of medical error (e.g., wrong-site surgery in neurosurgery); (d) studies focusing on a particular group of employees where generalisability to other groups would not be feasible (i.e., only nurses, physicians, pharmacists); (e) conference abstracts, narrative reviews, editorial and other types of studies but systematic reviews; (f) studies related to adverse events only; and (g) studies with no effect on medical errors.

### Search strategy

To identify relevant interventions, we searched the four databases (PubMed, Scopus, Ovid Medline and Embase) from Oct 1977 until January 2021 and selected English-only publications. Multiple keywords related to medical errors were researched and customized for each database. We used the filters for searching papers on interventions to reduce medical error to maximize the sensitivity of our literature search. We did not make any limitations on the outcomes. Additionally, references from the included systematic reviews were checked and added to selected studies. Our search strategy was adjusted for each database accordingly. For example, following combination was used for Pubmed database: ((((((((((((((((medical errors[MeSH Terms] OR “recording error”[Title/Abstract]) OR “no harm”[Title/Abstract] OR “patient fall^*^”[Title/Abstract]) OR “hospital infection”[Title/Abstract]) OR “transfusion error”[Title/Abstract]) OR “prescription error”[Title/Abstract]) OR “prescribing error”[Title/Abstract]) OR “CPR error”[Title/Abstract]))) OR “medication error”[Title/Abstract]) OR “near miss”[Title/Abstract]) OR “suicide”[Title/Abstract]) OR “sentinel event”[Title/Abstract]) OR “never event”[Title/Abstract]) AND systematic[sb]). An overview of the full search strategy can be found in Appendix 1.

### Data extraction

Two researchers independently extracted data from selected reviews. A third reviewer resolved any disagreements between the two reviewers. The following data were extracted: author, year, aim of the study, setting, medical error type, interventions, and the overall results if reported. Only reviews that met our selection criteria were extracted and analyzed.

### Data analysis

The interventions of reviews were classified based on the medical error types. We additionally checked for the overlap between primary studies included in systematic reviews. Since there was no complete overlap between the reviews, none of the studies were excluded.

## Results

### Search results

**Figure 1:**
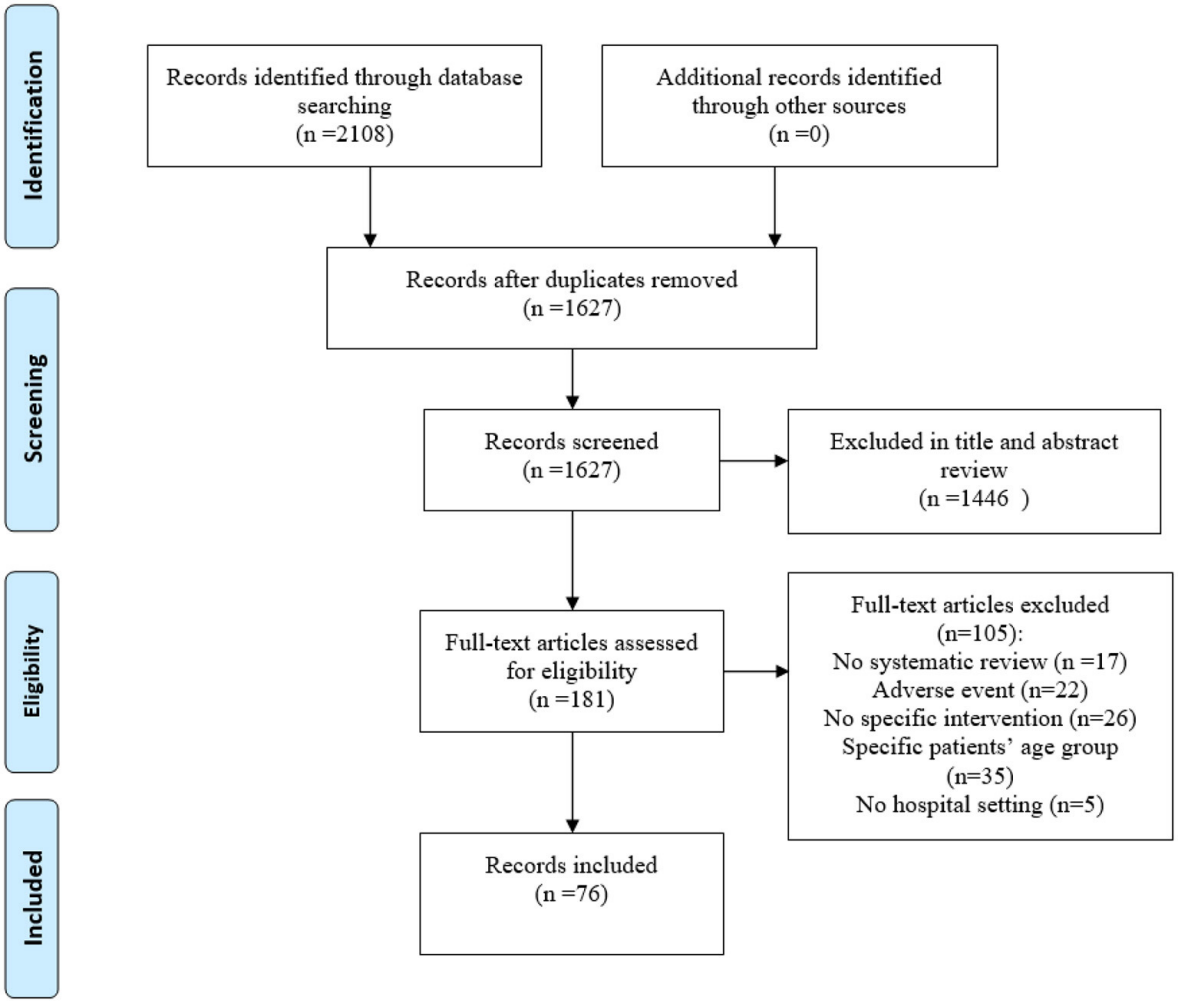
PRISMA flow diagram for the review process.

The initial search provided 2108 records (Figure 1). After eliminating duplicate papers, titles and abstract screening, 181 reviews underwent the full-text assessment. In total 76 reviews met the inclusion criteria, 105 were excluded for various reasons (Figure 1).

### Characteristics of the included systematic reviews

More than half of systematic reviews (67%) were published between 2013 and 2020 (*n* = 51). 66% of reviews were about medication error (*n* = 49), and 74% were related to all hospital settings (*n* = 56). The included studies reported on interventions for eight types of medical errors: overall medical error (13 interventions), medication error (37 interventions), patients' fall (11 interventions), healthcare-associated infections (21 interventions), diagnostic errors (7 interventions), transfusion and testing errors (8 interventions), surgical errors (3 interventions), and patients' suicide (13 interventions) (Table 1). Table 2 provides an overview of the impact of interventions on medical error reduction by intervention group. A more detailed overview of the impact of studies, including their aim, setting, and overall results can be found in Supplementary Table 1.

**Table 1:** Interventions to reduce medical error by medical error category.

**Medical error category**	**Interventions groups**	**Number of interventions**
Overall medical error (1–10)	Use of electronic systems Process interventions Patient-centered intervention Inter-professional education	7 4 1 1
Medication error (4, 5, 7, 11–57)	Use of electronic systems Pharmacists and clinical pharmacist role Process interventions Leadership or managerial manners and strategies Smart pumps impact	10 1 19 6 1
Patients' fall (5, 58–62)	Education and professional skills Methods/tools evaluating patients' fall risk Process and patient care programs Hourly rounding programs Organizational and workplace culture	3 3 3 1 1
Healthcare-associated infections (18, 21, 42, 58, 61, 63–69)	Caregivers' education and behavioral change interventions Process interventions Managerial and organizational interventions Use of medication interventions Environment/equipment cleaning	4 8 5 3 1
Diagnostic errors (5, 70, 71)	Digital and electronic interventions Patient identification and checking Quality improvement methodologies	3 2 2
Transfusion and testing errors (72, 73)	Identification of patients (labeling and barcoding)	8
Surgical errors (18, 42, 74, 75)	Use of checklists and counting materials Use of radio-frequency identification technology	2 1
Patients' suicide (76, 77)	Measures to reduce absconding and engagement with patient's family Contact interventions Process and patient care programs	2 3 8

**Table 2:** Impact of interventions on medical error reduction by intervention group.

	**Overall medical error**	**Medication error**	**Patients' fall**	**Healthcare-associated infections**	**Diagnostic error**	**Transfusion and testing errors**	**Surgical errors**	**Patients' suicide**
Caregivers' education and behavioral change interventions				+ + 2 reviews (58, 64) + 2 reviews (65, 69)				
Digital and electronic					++1 review (5) + 1 review (70)			
Education and professional skills			+ + 2 reviews (58, 59) + 1 review (60)					
Use of electronic systems	+ + 2 reviews (3, 4) + 2 reviews (1, 2)	+ + 12 reviews (4, 22, 24–31, 56, 57) + 13 reviews (5, 34, 35, 44–53)						
Environment/equipment cleaning				++ 1 review (63)				
Identification of patients (labeling and barcoding)						++ 1 review (72) +1 review (73)		
Inter-professional education	+ + 1 review (9)							
Leadership or managerial manners and strategies		+ + 4 reviews (14, 17, 22, 23) + 1 review (21)						
Managerial and organizational interventions				++1 review (64) + 3 reviews (21, 61, 65)				
Measures to reduce absconding and engagement with patient's family								+ 1 review (76)
Methods/tools evaluating patients' fall risk			+ +1 review (59) + 1 review (5)					
Organizational and workplace cultures			+ 1 review (61)					
Patient identification					+ 1 review (70)			
Patient-centered interventions	+ 1 review (8)							
Pharmacists and clinical pharmacist role		++ 6 reviews (37–41, 43) + 7 reviews (5, 32, 33, 35, 36, 42)						
Use of checklists and counting materials							++ 2 review (18, 75) +1 review (42)	
Process and patient care interventions			++1 review (59)					++1 review (77)
Process interventions	++ 1 review (7) + 3 review s (5, 6, 10)	++ 9 reviews (7, 13–20) + 4 reviews (5, 11, 12, 55)		++ 1 review (18) +2 reviews (42, 66)				
Quality improvement methodologies					+1 review (71)			
Smart pumps impact		+ 2 review (5, 54)						
Radio-frequency identification technology							+1 review (74)	
Contact interventions								++1 review (77)
Use of medication				++ 1 review (67) + 2 reviews (42, 68)				
Hourly rounding programs			++ 1 review (62)					

++ effective in reduction / significant reduction, + some evidence of reduction.

### Interventions based on medical error types

#### Overall medical error

This group of interventions was not restricted to a specific medical error type. It included four interventions groups (i.e., use of electronic systems, patient-centered intervention, process interventions, and inter-professional education). In total, ten reviews focused on overall medical errors (28–37) and included 257 primary studies (Table 1). Five reviews focused on the use of electronic systems to reduce overall medical error levels using health information systems, computerized provider order entry systems combined with clinical decision support systems, diagnostic and clinical decision-making aids, error-resistant systems, computer-enabled discharge communication, personal digital assistants, human simulation training) (28–32). Four reviews presented the process interventions such as failure mode and effects analysis, proactive technique, systematic safety processes, teamwork and communication training interventions, and reactive systematic safety processes in reducing risks, medical errors and adverse events (32–34, 37). One study referred to a patient-centered intervention, i.e., documentation through patient involvement and feedback on the medical file (35). Reeves et al. focused on interprofessional education (36) (Supplementary Table 1).

Reviews confirmed that using electronic systems could reduce (28, 29) or effectively and significantly (30, 31) reduce medical errors. For example, Charles et al. (29) stated that computerized provider order entry reduces medical error and adverse drug events. The effect would be more when combined with clinical decision support systems to alert healthcare providers of medical errors (29). Studies that focused on other intervention groups [i.e., process interventions (32–34, 37), patient-centered intervention (35), and inter-professional education (36)] presented some evidence of their potential to reduce medical errors (Table 2). For example, using process interventions minimizes risks and improves service quality (33). In contrast, interprofessional education could reduce medical errors and enhance behavior culture in the emergency department (36).

#### Medication error

This intervention group related to medication errors and specific subcategories (prescribing, dispensing, administering, transcription and dose errors). These interventions fell into five groups: use of electronic systems, pharmacists and clinical pharmacist involvement in the treatment process, process interventions, leadership or managerial manners, and strategies and smart pumps impact. Overall, 49 reviews focused on interventions to reduce medication errors. This was the most prominent intervention category, including 1,380 primary studies (Table 1). Twentyfive reviews focused on using electronic systems (14, 16, 31, 32, 38–58). Twelve reviews focused on pharmacists and clinical pharmacist involvement in the treatment process (13, 17, 32, 41, 59–66). Five reviews presented leadership or managerial manners interventions (12, 56, 67–69). The remaining 12 reviews stated process interventions (9, 12, 32, 34, 67, 70–76), and two reviews focused on smart pumps impact (32, 77) (Table 2).

Similarly to overall medical error interventions, reviews focusing on electronic systems provided evidence that they could reduce (14, 16, 32, 38–48) or effectively and significantly (31, 49–58) reduce medication errors. For example, the most significant results were noted for computerized provider order entry in 96% error interception and 90% reduction of medication errors (41, 44). There was evidence that leadership or managerial manners intervention could effectively and significantly reduce medication errors (12, 56, 67–69). For example, redesign of diabetes prescribing charts incorporating prescribing guidelines, diabetes prescription error management pathway, and mandatory e-learning reduced insulin prescription errors from 65 to 2% (67) (Table 2, Supplementary Table 1). Reviews on pharmacists and clinical pharmacist involvement in the treatment process presented evidence of some to a very effective and significant reduction on medical errors. For example, pharmacists' participation in medical treatment leads to a 43% reduction in prescribing errors and a 27% reduction in overall medication errors (63, 64). Most reviews on process interventions had also shown that such intervention could effectively and significantly reduce medication errors (9, 12, 34, 67, 70–74), with only a few (32, 75, 76, 78) presenting only some evidence of medication error reduction. For example, double-checking reduce medication error from 2.98 to 2.12 per 1,000 medication administered and dispensing error from 9.8 to 6 (73).

#### Patients' fall

This group of interventions focused on interventions that could reduce patients' falls by using four different categories of interventions (professional skills and education, methods/tools evaluating patients' fall risk, process and patient care programs, organizational and workplace culture). In total, six reviews (10, 26, 27, 32, 79, 80) focused on fall prevention and included 14 primary studies. Three reviews focused on using education and professional skills interventions (10, 27, 79). Two reviews presented using methods and tools evaluating patients' fall risk (27, 32). Cumbler et al. reported process and patient care programs as beneficial interventions (27). One study focused on hourly rounding programs (80), and Braithwaite et al. presented organizational and workplace culture interventions (26) (Table 2).

Based on the results of reviews, education and professional skills interventions effectively reduced or led to a significant reduction in patients' falls (10, 27, 80), while another review showed some evidence of a reduction in patients' falls (79). For example, there were patients' fall differences in intervention groups vs. control groups through patient-centered interventions (180 in intervention group vs. 319 in control group) (79). There was evidence that methods/tools evaluating patients' fall risk intervention could effectively and significantly reduce medical errors (27), and other reviews showed that could reduce patients' falls (32). For example, using the Morse fall scale decreased falls (27). Two remaining studies focused on effectively and significantly reducing patients' falls (27, 80), and the other had some evidence of reduction (26). For example, staff education, care planning, patient training in rehabilitation and nutritionist support lead to a reduction in falls from 16.28 to 6.29 per 1,000 patient days (27) (Table 2, Supplementary Table 1).

### Healthcare-associated infections

Twelve reviews and 382 primary studies focused on five groups of interventions that could reduce healthcare-associated infections (caregivers' educational and behavioral change interventions, process interventions, managerial and organizational interventions, using medication interventions and environment/equipment cleaning) (Table 1). Four reviews focused on the caregivers' education and behavioral changes (10, 81–83). Three reviews focused on process interventions (65, 72, 84). Four reviews presented the managerial and organizational interventions (26, 69, 81, 83). Three reviews reported medication interventions (65, 85, 86). Schabrun et al. focused on equipment cleaning (87) (Table 2).

Caregivers' education and behavioral change effectively reduced healthcare-associated infections (10, 81), and the other two reviews showed some evidence of a reduction in healthcare-associated infections (82, 83). For example, hand-hygiene campaigns reduced nosocomial infection rates (median effect 49%) (81). Boyd et al. presented an effective or significant reduction in healthcare-associated infections (72), and two reviews showed that these interventions could reduce healthcare-associated infections (65, 84). For example, the Keystone intensive care unit intervention for central line-associated bloodstream infections and chlorhexidine for vascular catheter site care economically reduced healthcare-associated infections (65). One review stated that managerial and organizational interventions are significant or effective in reducing healthcare-associated infections (81), while three studies have some evidence on reducing healthcare-associated infections (26, 69, 83). For example, antibiotic stewardship, antibiotic restriction, guidelines, education, and performance feedback showed a significant decrease ranging from 13 to 82% (81). One review of medication interventions reported a significant decline (28%) in surgical site infection using a chlorhexidine impregnated dressing that applied to the surgical site (86). Another review demonstrated an effective reduction (82.1%) in colony-forming units after cleaning pieces of equipment with alcohol (87).

### Diagnostic error

Three studies that included 68 primary studies focused on three intervention categories (digital and electronic interventions, patient identification and checking and quality improvement methodologies) that affect diagnostic errors (2, 32, 88) (Table 1). Two studies presented the use of digital and electronic interventions (2, 32). One study focused on the use of patient identification (2). Amaratunga et al. focused on quality improvement methodologies (88). One review focused on digital and electronic interventions showed a significant effect of interventions to reduce diagnostic error. The other one presented some evidence of diagnostic error reduction (2, 32). For example, clinical decision support systems and a web-based diagnostic reminder system significantly reduced diagnostic errors (32). Zhou et al. (2) presented some evidence of a reduction in diagnostic error using patient identification. For example, the patient identification check, obtaining informed consent, verifying the correct side and site, and a final check by the radiologist decreased the incidence rate of diagnostic error from 0.03% (9 of 32,982) to 0.005% (2). Another review reported some evidence of a reduction in diagnostic error within radiology by lean and Six Sigma approaches as quality improvement methodologies (88).

### Transfusion and testing errors

Two reviews included 26 primary studies focused on the identification of patients (labeling and barcoding) intervention (11, 89) (Table 1). The results of Snyder et al.'s review was effective in reducing transfusion and testing errors (89), and another review showed some evidence on reducing transfusion and testing errors (11) (Table 2). For example, labeling significantly reduces testing errors, so the most effective intervention in reducing transfusion and testing errors was barcoding systems, which reduced 2.26 errors to 0.17 errors per 10,000 specimens (89).

### Surgical errors

Four reviews included 38 primary studies focused on two intervention groups to reduce surgical errors (use of checklists and counting instruments and material and use of radio-frequency identification technology) (15, 65, 72, 90) (Table 1). Three reviews reported using checklists and counting materials interventions (65, 72, 90). Another review focused on radio-frequency identification technology (15) (Table 2). Two reviews showed an effective reduction in surgical errors (72, 90) while, Etchells et al.'s review had some evidence related to reducing surgical errors (65). For example, using checklists (or similar interventions) could reduce equipment errors in the operating room by 48.6% (90). One review showed some evidence to reduce retained surgical instrument errors, reduce the risk of counting errors, and improve workflow using radio-frequency identification technology (15) (Table 2).

### Patients' suicide

Two reviews included 112 primary studies focused on reducing patients' suicide (91, 92) (Table 1). One review focused on reducing absconding and engagement with patient's family intervention (91). Doupnik et al., focused on process and patient care interventions and contact interventions (92) (Table 2). Bowers et al. reported measures to reduce absconding and engagement with patient's family intervention, showed some evidence to reduce absconding without locking the door and engage with patients' family problems to reduce patients' suicide (91). Another review focused on process, and patient care interventions and contact interventions showed significant reduction (pooled odds ratio, 0.69) in patients' suicide by using 11 interventions (i.e., telephone, postcard, letters, coordination between the mental health care team, and follow up mental health care team) (92) (Supplementary Table 1, Table 2).

## Discussion

We systematically reviewed systematic reviews for interventions to reduce medical errors in hospitals. Studies related to preventing medication errors included approximately 35 interventions. We identified 21 groups of interventions falling into seven broader categories of medical errors. The least studied category of medical errors was related to patients' suicide and surgical errors. Our findings showed that among 101 presented interventions, the use of electronic systems intervention group, was included in most of the reviews (27 reviews). This group included interventions that reduce medication and overall medical errors. Most interventions were related to the processing group (30 interventions). Also, this group had three types of errors (overall medical error, medication error, and healthcare-associated infections). The most effective interventions were related to medication errors among medical error types (27 reviews) and electronic systems among intervention groups (12 reviews).

Patient safety has several requirements such as safe infrastructure, technologies and medical devices, patient and staff education, information, professional participation in patient safety programs, and ensuring that all individuals receive secure health services, regardless of where they are delivered. This was reiterated in the resolution on “Global action on patient safety” in May 2019 (WHA72.6) (93). In particular, the resolution requests the World Health Organization's Director-General to formulate a global patient safety action plan in consultation with the Member States, regional economic integration organizations and all relevant stakeholders, including in the private sector. As stated in the resolution, to achieve the highest level of patient safety and to be able to reduce medical error and adverse events, one needs to recognize patient safety as a health priority in health sector policies and programs, collaborate with other member states along with the improvement of national policies, programs, guidelines, strategies and tools.

There are several ways, policies and procedures to identify medical errors. Differences in error identification methods affect the incidence of errors and error reduction interventions. These methods include voluntary reporting, direct observation, patient and family reporting, and retrospective and prospective methods (cohort and cross sectional studies) and related techniques (e.g., failure mode, effects analysis, and root cause analysis) (94–99).

The most effective interventions related to patient satisfaction referred to managerial and process interventions that show patients do not have enough knowledge about medical issues. Process and administrative interventions increase their satisfaction as a perceived issue (70, 80). Effective interventions to reduce costs and increase efficiency were related to using electronic systems and processes and managerial or leadership strategies (9, 12, 54, 70). For example, electronic distribution drug systems decreased by €44,295 in a month (9). Effective interventions related to reducing death referred to the use of electronic systems and process interventions (16, 70). For example, commercial computerized provider order entry led to a 12% reduction in intensive care units mortality rates (16). Effective interventions for increasing health care quality were referred to as checklists and counting materials, environment/equipment cleaning, use of electronic systems, and process interventions (9, 54, 87, 90). Effective interventions related to patient safety were associated with the use of electronic systems, process, education and professional skills, methods/tools evaluating patients' fall risk, and process and patient care interventions groups (9, 27, 34, 51, 53, 58).

As we highlighted in our study findings, use of electronic systems has a wide effect on reduction of medical errors and related deaths, efficiency and effectiveness of services, and improvement of patient safety. Of course, when using electronic systems, like any other method, one must pay attention to its specific limitations and considerations. For example, implementation of computerized prescription order entry can lead to wrong drug selection from drop-down menus (49). Nonetheless, computerized prescription order entry systems are more effective to detect medical errors when they are bundled with clinical decision support systems, which has the potential to prevent errors of medication forms nearly completely (29, 100). Simulation systems prevent iatrogenic risk related to medication errors, if the program is well designed (14).

Our review has several limitations. One is that medical errors cover a very wide range of topics that cannot be addressed in one review article. For example, topics that were left outside the scope of this paper include error identification policies, procedures and methods, disclosure approaches, and incidence of medical errors. Another limitation is that we focused on the interventions in the hospital settings. Due to the high number of papers related to the effect of interventions on medical error, we restricted our analysis to documents that reported the positive impact of the intervention on medical error reduction. Also, our study was limited to systematic reviews that had different focus; hence, meta-analyses were not possible.

## Conclusion

Prevention of medical errors is vital in reducing patient's harm and improving overall patient outcomes. A review of the combined evidence of 73 systematic reviews found that a wide range of interventions could be used to prevent and decrease of incidence of medical errors. Process and managerial interventions, and use of electronic systems had a critical role in medical error reduction.

## Data availability statement

The original contributions presented in the study are included in the article/Supplementary material, further inquiries can be directed to the corresponding author/s.

## Author contributions

EA-E and LD conceived the basic and original idea, outlined the study, literature review, data acquisition, data analysis, interpretation of data, and drafted the article. VS contributed to data acquisition, data analysis, interpretation of data, and drafting and revising of manuscript. All authors participated in the final design, revision of the manuscript, and have read and approved the manuscript.

## Conflict of interest

The authors declare that the research was conducted in the absence of any commercial or financial relationships that could be construed as a potential conflict of interest.

## Publisher's note

All claims expressed in this article are solely those of the authors and do not necessarily represent those of their affiliated organizations, or those of the publisher, the editors and the reviewers. Any product that may be evaluated in this article, or claim that may be made by its manufacturer, is not guaranteed or endorsed by the publisher.

## References

[1] Donaldson MS Corrigan JM Kohn LT. To Err is Human: Building a Safer Health System. Vol. 6. Washington, DC: National Academies Press (2000). 25077248

[2] Zhou Y Boyd L Lawson C. Errors in medical imaging and radiography practice: a systematic review. J Med Imag Radiat Sci. (2015) 46:435–41. 10.1016/j.jmir.2015.09.002 31052125

[3] Heideveld-Chevalking A Calsbeek H Griffioen I Damen J Meijerink W Wolff A. Development and validation of a Self-assessment Instrument for Perioperative Patient Safety (SIPPS). BJS open. (2018) 2:381–91. 10.1002/bjs5.82 30511039 PMC6254004

[4] Andel C Davidow SL Hollander M Moreno DA. The economics of health care quality and medical errors. J Health Care Finance. (2012) 39:39. 23155743

[5] Patient safety, Data and statisics: WHO Regional office for Europe. (2021). Available online at: https://www.euro.who.int/en/health-topics/Health-systems/patient-safety/data-and-statistics (accessed January 29, 2021).

[6] Patient safety, Overview (2021). Available online at: https://www.who.int/health-topics/patient-safety#tab=tab_1 (accessed January 29, 2021).

[7] Sreeramoju P. Preventing healthcare-associated infections: Beyond best practice. Am J Med Sci. (2013) 345:239–44. 10.1097/MAJ.0b013e31824435e6 22270397

[8] Scott RD. The direct medical costs of healthcare-associated infections in US hospitals and the benefits of prevention. (2009).

[9] Ahtiainen HK Kallio MM Airaksinen M Holmström AR. Safety, time and cost evaluation of automated and semi-automated drug distribution systems in hospitals: a systematic review. Eur J Hospital Pharmacy. (2019). 10.1136/ejhpharm-2018-001791 32839256 PMC7447254

[10] Coelho P. Relationship between nurse certification and clinical patient outcomes: a systematic literature review. J Nurs Care Qual. (2019). 10.1097/NCQ.0000000000000397 30817408

[11] Cottrell S Watson D Eyre TA Brunskill SJ Dorée C Murphy MF. Interventions to reduce wrong blood in tube errors in transfusion: A systematic review. Transfus Med Rev. (2013) 27:197–205. 10.1016/j.tmrv.2013.08.003 24075096

[12] Mikrani R Naveed M Mikrani A Yasmeen S Akabar M Xiaohui Z. The impact of clinical pharmacy services in Nepal in the context of current health policy: a systematic review. J Public Health. (2019). 10.1007/s10389-019-01042-y

[13] Noormandi A Karimzadeh I Mirjalili M Khalili H. Clinical and economic impacts of clinical pharmacists' interventions in Iran: a systematic review. DARU, J Pharmaceutical Sci. (2019) 27:361–78. 10.1007/s40199-019-00245-8 30674033 PMC6593130

[14] Sarfati L Ranchon F Vantard N Schwiertz V Larbre V Parat S . Human-simulation-based learning to prevent medication error: A systematic review. J Eval Clin Pract. (2019) 25:11–20. 10.1111/jep.12883 29383867

[15] Schnock KO Biggs B Fladger A Bates DW Rozenblum R. Evaluating the impact of radio frequency identification retained surgical instruments tracking on patient safety: literature review. J Patient Saf. (2017). 10.1097/PTS.0000000000000365 28230583

[16] Prgomet M Li L Niazkhani Z Georgiou A Westbrook JI. Impact of commercial computerized provider order entry (CPOE) and clinical decision support systems (CDSSs) on medication errors, length of stay, and mortality in intensive care units: A systematic review and meta-analysis. J Am Med Inform Assoc. (2017) 24:413–22. 10.1093/jamia/ocw145 28395016 PMC7651905

[17] Cohen V Jellinek SP Hatch A Motov S. Effect of clinical pharmacists on care in the emergency department: A systematic review. Am J Health-Syst Pharmacy. (2009) 66:1353–61. 10.2146/ajhp080304 19635771

[18] Lucas AJ. Improving medication safety in a neonatal intensive care unit. Am J Health-Syst Pharm. (2004) 61:33–7. 10.1093/ajhp/61.1.33 14725118

[19] Lee SE Quinn BL. Incorporating medication administration safety in undergraduate nursing education: a literature review. Nurse Educ Today. (2019) 72:77–83. 10.1016/j.nedt.2018.11.004 30453203

[20] Owoc J Manczak M Tombarkiewicz M Robert Olszewski R. Association between physician burnout and self-perceived error: Meta-analysis. Eur J Prev Cardiol. (2019) 26:S57. 10.1177/2047487319860048 34951608

[21] Mehtsun WT Ibrahim AM Diener-West M Pronovost PJ Makary MA. Surgical never events in the United States. Surgery. (2013) 153:465–72. 10.1016/j.surg.2012.10.005 23257079

[22] Omar I Graham Y Singhal R Wilson M Madhok B Mahawar KK. Identification of common themes from never events data published by NHS England. World J Surg. (2021) 45:697–704. 10.1007/s00268-020-05867-7 33216170

[23] Kim CW Myung SJ Eo EK Chang Y. Improving disclosure of medical error through educational program as a first step toward patient safety. BMC Med Educ. (2017) 17:1–6. 10.1186/s12909-017-0880-9 28259161 PMC5336642

[24] Wallis J Fletcher D Bentley A Ludders J. Medical errors cause harm in veterinary hospitals. Front Veter Sci. (2019) 6:12. 10.3389/fvets.2019.00012 30805349 PMC6370638

[25] Benimana C Small M Rulisa S. Preventability of maternal near miss and mortality in Rwanda: a case series from the University Teaching Hospital of Kigali (CHUK). PLoS ONE. (2018) 13:e0195711. 10.1371/journal.pone.0195711 29944664 PMC6019403

[26] Braithwaite J Herkes J Ludlow K Testa L Lamprell G. Association between organisational and workplace cultures, and patient outcomes: Systematic review. BMJ Open. (2017) 7:e017708. 10.1136/bmjopen-2017-017708 29122796 PMC5695304

[27] Cumbler EU Simpson JR Rosenthal LD Likosky DJ. Inpatient falls: defining the problem and identifying possible solutions. Part I: An Evidence-Based Review. The Neurohospitalist. (2013) 3:135–43. 10.1177/1941874412470665 24167647 PMC3805440

[28] Ahmadian L Salehi Nejad S Khajouei R. Evaluation methods used on health information systems (HISs) in Iran and the effects of HISs on Iranian healthcare: A systematic review. Int J Med Inform. (2015) 84:444–53. 10.1016/j.ijmedinf.2015.02.002 25746766

[29] Charles K Cannon M Hall R Coustasse A. Can utilizing a computerized provider order entry (CPOE) system prevent hospital medical errors and adverse drug events? Perspect Health Inf Manage. (2014) 11:1b. 25593568 PMC4272436

[30] Motamedi SM Posadas-Calleja J Straus S Bates DW Lorenzetti DL Baylis B . The efficacy of computer-enabled discharge communication interventions: A systematic review. BMJ Quality and Safety. (2011) 20:403–15. 10.1136/bmjqs.2009.034587 21262793

[31] Prgomet M Georgiou A Westbrook JI. The Impact of Mobile Handheld Technology on Hospital Physicians' Work Practices and Patient Care: A Systematic Review. J Am Med Inform Assoc. (2009) 16:792–801. 10.1197/jamia.M3215 19717793 PMC3002124

[32] Dückers M Faber M Cruijsberg J Grol R Schoonhoven L Wensing M. Safety and risk management interventions in hospitals: A systematic review of the literature. Medical Care Research and Review. (2009) 66:90S–119S. 10.1177/1077558709345870 19759391

[33] Asgari Dastjerdi H Khorasani E Yarmohammadian MH Ahmadzade MS. Evaluating the application of failure mode and effects analysis technique in hospital wards: a systematic review. J Injury Violence Res. (2017) 9:51. 10.5249/jivr.v9i1.794 28039688 PMC5279992

[34] Damiani G Pinnarelli L Scopelliti L Sommella L Ricciardi W. A review on the impact of systematic safety processes for the control of error in medicine. Med Sci Monitor. (2009) 15:RA157–RA66. 19564841

[35] Vermeir P Degroote S Vandijck D Van Tiggelen H Peleman R Verhaeghe R . The patient perspective on the effects of medical record accessibility: a systematic review. Acta Clinica Belgica: Int J Clin Laboratory Med. (2017) 72:186–94. 10.1080/17843286.2016.1275375 28056665

[36] Reeves S Perrier L Goldman J Freeth D Zwarenstein M. Interprofessional education: Effects on professional practice and healthcare outcomes (update). Cochrane Datab System Rev. (2013) 2013:CD002213. 10.1002/14651858.CD002213.pub3 23543515 PMC6513239

[37] Alsabri M Boudi Z Lauque D Roger DD Whelan JS Östlundh L . Impact of teamwork and communication training interventions on safety culture and patient safety in emergency departments: a systematic review. J Patient Saf. (2020). 10.1097/PTS.0000000000000782 33890752

[38] Eslami S de Keizer NF Abu-Hanna A. The impact of computerized physician medication order entry in hospitalized patients–a systematic review. Int J Med Inform. (2008) 77:365–76. 10.1016/j.ijmedinf.2007.10.001 18023611

[39] Khajouei R JasperS MWM. The impact of CPOE medication systems' design aspects on usability, workflow and medication orders a systematic review. Methods Inf Med. (2010) 49:3–19. 10.3414/ME0630 19582333

[40] Ammenwerth E Schnell-Inderst P Machan C Siebert U. The effect of electronic prescribing on medication errors and adverse drug events: a systematic review. J Am Med Inform Assoc. (2008) 15:585–600. 10.1197/jamia.M2667 18579832 PMC2528040

[41] Acheampong F Anto BP Koffuor GA. Medication safety strategies in hospitals - A systematic review. Int J Risk Safety Med. (2014) 26:117–31. 10.3233/JRS-140623 25214157

[42] Kaushal R Shojania KG Bates DW. Effects of computerized physician order entry and clinical decision support systems on medication safety: A systematic review. Arch Intern Med. (2003) 163:1409–16. 10.1001/archinte.163.12.1409 12824090

[43] Manias E Kusljic S Wu A. Interventions to reduce medication errors in adult medical and surgical settings: a systematic review. Ther Adv Drug Saf. (2020) 11:2042098620968309. 10.1177/2042098620968309 33240478 PMC7672746

[44] Shitu Z Aung MMT Kamauzaman THT Bhagat V Rahman AFA. Medication error in hospitals and effective intervention strategies: A systematic review. Res J Pharm Technol. (2019) 12:4669–77. 10.5958/0974-360X.2019.00804.7

[45] Keers RN Williams SD Cooke J Walsh T Ashcroft DM. Impact of interventions designed to reduce medication administration errors in hospitals: A systematic review. Drug Safety. (2014) 37:317–32. 10.1007/s40264-014-0152-0 24760475

[46] Jia P Zhang L Chen J Zhao P Zhang M. The effects of clinical decision support systems on medication safety: An overview. PLoS ONE. (2016) 11:e0167683. 10.1371/journal.pone.0167683 27977697 PMC5157990

[47] Pawloski PA Brooks GA Nielsen ME Olson-Bullis BA. A systematic review of clinical decision support systems for clinical oncology practice. JNCCN J National Comprehensive Cancer Network. (2019) 17:331–8. 10.6004/jnccn.2018.7104 30959468 PMC6563614

[48] Bouaud J Lamy JB Section Editors for the IYSoDS. A medical informatics perspective on clinical decision support systems. Findings from the yearbook 2013 section on decision support. Yearbook of Med Inform. (2013) 8:128–31. 10.1055/s-0038-1638844 23974560

[49] Velez-Diaz-Pallares M Perez-Menendez-Conde C Bermejo-Vicedo T. Systematic review of computerized prescriber order entry and clinical decision support. Am J Health Syst Pharm. (2018) 75:1909–21. 10.2146/ajhp170870 30463867

[50] Nuckols TK Smith-Spangler C Morton SC Asch SM Patel VM Anderson LJ . The effectiveness of computerized order entry at reducing preventable adverse drug events and medication errors in hospital settings: A systematic review and meta-analysis. System Rev. (2014) 3:56. 10.1186/2046-4053-3-56 24894078 PMC4096499

[51] Ahmed Z Garfield S Jani Y Jheeta S Franklin BD. Impact of electronic prescribing on patient safety in hospitals: Implications for the UK. Clin Pharmacist. (2016) 8:1–11. 10.1211/CP.2016.20201013

[52] Roumeliotis N Sniderman J Adams-Webber T Addo N Anand V Rochon P . Effect of Electronic Prescribing Strategies on Medication Error and Harm in Hospital: a Systematic Review and Meta-analysis. J Gen Intern Med. (2019) 34:2210–23. 10.1007/s11606-019-05236-8 31396810 PMC6816608

[53] Ojeleye O Avery A Gupta V Boyd M. The evidence for the effectiveness of safety alerts in electronic patient medication record systems at the point of pharmacy order entry: A systematic review. BMC Med Inform Decis Making. (2013) 13:1–10. 10.1186/1472-6947-13-69 23816138 PMC3702525

[54] Chaudhry B Wang J Wu S Maglione M Mojica W Roth E . Systematic review: Impact of health information technology on quality, efficiency, and costs of medical care. Ann Intern Med. (2006) 144:742–52. 10.7326/0003-4819-144-10-200605160-00125 16702590

[55] Devin J Cleary BJ Cullinan S. The impact of health information technology on prescribing errors in hospitals: a systematic review and behaviour change technique analysis. Syst Rev. (2020) 9:275. 10.1186/s13643-020-01510-7 33272315 PMC7716445

[56] Yang C Yang L Xiang X Tang Y Wang H Bobai N . Interventions assessment of prescription automatic screening system in chinese hospitals: a systematic review. Drug Inf J. (2012) 46:669–76. 10.1177/0092861512454417

[57] Schedlbauer A Prasad V Mulvaney C Phansalkar S Stanton W Bates DW . What evidence supports the use of computerized alerts and prompts to improve clinicians' prescribing behavior? J Am Med Inform Assoc. (2009) 16:531–8. 10.1197/jamia.M2910 19390110 PMC2705257

[58] Alanazi A Alomar M Aldosari H Shahrani A Aldosari B. The effect of electronic medication administration records on the culture of patient safety: a literature review. Studies Health Technol Inform. (2018) 251:223–6. 10.3233/978-1-61499-880-8-223 29968643

[59] Poh EW McArthur A Stephenson M Roughead EE. Effects of pharmacist prescribing on patient outcomes in the hospital setting: A systematic review. JBI Datab System Rev Implement Reports. (2018) 16:1823–73. 10.11124/JBISRIR-2017-003697 30204671

[60] Bethishou L Herzik K Fang N Abdo C Tomaszewski DM. The impact of the pharmacist on continuity of care during transitions of care: A systematic review. J Am Pharm Assoc. (2020) 60:163–77.e2. 10.1016/j.japh.2019.06.020 31375332

[61] Mekonnen AB McLachlan AJ Brien JAE. Pharmacy-led medication reconciliation programmes at hospital transitions: A systematic review and meta-analysis. J Clin Pharm Ther. (2016) 41:128–44. 10.1111/jcpt.12364 26913812

[62] Chiewchantanakit D Meakchai A Pituchaturont N Dilokthornsakul P Dhippayom T. The effectiveness of medication reconciliation to prevent medication error: A systematic review and meta-analysis. Res Social Adm Pharm. (2020) 16:886–94. 10.1016/j.sapharm.2019.10.004 31607507

[63] Wang T Benedict N Olsen KM Luan R Zhu X Zhou N . Effect of critical care pharmacist's intervention on medication errors: a systematic review and meta-analysis of observational studies. J Crit Care. (2015) 30:1101–6. 10.1016/j.jcrc.2015.06.018 26260916

[64] Naseralallah LM Hussain TA Jaam M Pawluk SA. Impact of pharmacist interventions on medication errors in hospitalized pediatric patients: a systematic review and meta-analysis. Int J Clin Pharm. (2020) 42:979–94. 10.1007/s11096-020-01034-z 32328958

[65] Etchells E Koo M Daneman N McDonald A Baker M Matlow A . Comparative economic analyses of patient safety improvement strategies in acute care: a systematic review. BMJ Quality Safety. (2012) 21:448–56. 10.1136/bmjqs-2011-000585 22523319

[66] Gillani SW Gulam SM Thomas D Gebreigziabher FB Al-Salloum J Assadi RA . Role and services of pharmacist in the prevention of medication errors: a systematic review. Curr Drug Saf. (2020). 10.2174/1574886315666201002124713 33006539

[67] Bain A Hasan SS Babar ZUD. Interventions to improve insulin prescribing practice for people with diabetes in hospital: a systematic review. Diabetic Med. (2019) 36:948–60. 10.1111/dme.13982 31050037

[68] Schroers G. Characteristics of interruptions during medication administration: An integrative review of direct observational studies. J Clin Nurs. (2018) 27:3462–71. 10.1111/jocn.14587 29945303

[69] Wong CA Cummings GG Ducharme L. The relationship between nursing leadership and patient outcomes: A systematic review update. J Nurs Manag. (2013) 21:709–24. 10.1111/jonm.12116 23865924

[70] Ashcraft S Bordelon C Fells S George V Thombley K Shirey MR. Interprofessional Clinical Rounding: Effects on Processes and Outcomes of Care. J Healthcare Quality. (2017) 39:85–94. 10.1097/JHQ.0000000000000039 27310299

[71] Wimpenny P Kirkpatrick P. Roles and systems for routine medication administration to prevent medication errors in hospital-based, acute care settings: a systematic review. JBI Libr Syst Rev. (2010) 8:405–46. 10.11124/jbisrir-2010-123 27820069

[72] Boyd JM Wu G Stelfox HT. The impact of checklists on inpatient safety outcomes: A systematic review of randomized controlled trials. J Hospital Med. (2017) 12:675–82. 10.12788/jhm.2788 28786436

[73] Alsulami Z Conroy S Choonara I. Double checking the administration of medicines: What is the evidence? A systematic review. Arch Dis Childhood. (2012) 97:833–7. 10.1136/archdischild-2011-301093 22550322

[74] McDowell SE Mt-Isa S Ashby D Ferner RE. Where errors occur in the preparation and administration of intravenous medicines: A systematic review and Bayesian analysis. Quality Safety Health Care. (2010) 19:341–5. 10.1136/qshc.2008.029785 20065297

[75] Jensen LS Merry AF Webster CS Weller CS Larsson L. Evidence-based strategies for preventing drug administration errors during anaesthesia. Anaesthesia. (2004) 59:493–504. 10.1111/j.1365-2044.2004.03670.x 15096243

[76] Ostini R Roughead EE Kirkpatrick CMJ Monteith GR Tett SE. Quality Use of Medicines - Medication safety issues in naming; Look-alike, sound-alike medicine names. Int J Pharm Pract. (2012) 20:349–57. 10.1111/j.2042-7174.2012.00210.x 23134093

[77] Ohashi K Dalleur O Dykes PC Bates DW. Benefits and risks of using smart pumps to reduce medication error rates: a systematic review. Drug Safety. (2014) 37:1011–20. 10.1007/s40264-014-0232-1 25294653

[78] Lehnbom EC Stewart MJ Manias E Westbrook JI. Impact of medication reconciliation and review on clinical outcomes. Ann Pharmacotherapy. (2014) 48:1298–312. 10.1177/1060028014543485 25048794

[79] Avanecean D Calliste D Contreras T Lim Y Fitzpatrick A. Effectiveness of patient-centered interventions on falls in the acute care setting compared to usual care: a systematic review. JBI Datab System Rev Implement Reports. (2017) 15:3006–48. 10.11124/JBISRIR-2016-003331 29219876

[80] Mitchell MD Lavenberg JG Trotta RL Umscheid CA. Hourly rounding to improve nursing responsiveness: a systematic review. J Nursing Administration. (2014) 44:462–72. 10.1097/NNA.0000000000000101 25148400 PMC4547690

[81] Murni I Duke T Triasih R Kinney S Daley AJ Soenarto Y. Prevention of nosocomial infections in developing countries, a systematic review. Paediatr Int Child Health. (2013) 33:61–78. 10.1179/2046905513Y.0000000054 23925279

[82] Safdar N Abad C. Educational interventions for prevention of healthcare-associated infection: A systematic review. Crit Care Med. (2008) 36:933–40. 10.1097/CCM.0B013E318165FAF3 18431283

[83] Aboelela SW Stone PW Larson EL. Effectiveness of bundled behavioural interventions to control healthcare-associated infections: a systematic review of the literature. J Hospital Infection. (2007) 66:101–8. 10.1016/j.jhin.2006.10.019 17320242

[84] Vonberg RP Gastmeier P. Hospital-acquired infections related to contaminated substances. J Hospital Infection. (2007) 65:15–23. 10.1016/j.jhin.2006.09.018 17145102

[85] Flanders SA Collard HR Saint S. Nosocomial pneumonia: State of the science. Am J Infect Control. (2006) 34:84–93. 10.1016/j.ajic.2005.07.003 16490612

[86] Savage JW Anderson PA. An update on modifiable factors to reduce the risk of surgical site infections. Spine J. (2013) 13:1017–29. 10.1016/j.spinee.2013.03.051 23711958

[87] Schabrun S Chipchase L. Healthcare equipment as a source of nosocomial infection: a systematic review. J Hospital Infection. (2006) 63:239–45. 10.1016/j.jhin.2005.10.013 16516340

[88] Amaratunga T Dobranowski J. Systematic review of the application of lean and six sigma quality improvement methodologies in radiology. J Am College Radiol. (2016) 13:1088–95.e7. 10.1016/j.jacr.2016.02.033 27209599

[89] Snyder SR Favoretto AM Derzon JH Christenson RH Kahn SE Shaw CS . Effectiveness of barcoding for reducing patient specimen and laboratory testing identification errors: A Laboratory Medicine Best Practices systematic review and meta-analysis. Clin Biochem. (2012) 45:988–98. 10.1016/j.clinbiochem.2012.06.019 22750145 PMC4518452

[90] Weerakkody RA Cheshire NJ Riga C Lear R Hamady MS Moorthy K . Surgical technology and operatingroom safety failures: A systematic review of quantitative studies. BMJ Quality and Safety. (2013) 22:710–8. 10.1136/bmjqs-2012-001778 23886892

[91] Bowers L Banda T Nijman H. Suicide inside: A systematic review of inpatient suicides. J Nervous Mental Disease. (2010) 198:315–28. 10.1097/NMD.0b013e3181da47e2 20458192

[92] Doupnik SK Rudd B Schmutte T Worsley D Bowden CF McCarthy E . Association of suicide prevention interventions with subsequent suicide attempts, linkage to follow-up care, and depression symptoms for acute care settings: a systematic review and meta-analysis. JAMA Psychiatry. (2020) 77:1021–30. 10.1001/jamapsychiatry.2020.1586 32584936 PMC7301305

[93] Kim JM Suarez-Cuervo C Berger Z Lee J Gayleard J Rosenberg C . Evaluation of patient and family engagement strategies to improve medication safety. Patient. (2018) 11:193–206. 10.1007/s40271-017-0270-8 28795338

[94] Garrouste-Orgeas M Philippart F Bruel C Max A Lau N Misset B. Overview of medical errors and adverse events. Ann Intensive Care. (2012) 2:2. 10.1186/2110-5820-2-2 22339769 PMC3310841

[95] Khammarnia M Ravangard R Barfar E Setoodehzadeh F. Medical Errors and Barriers to Reporting in Ten Hospitals in Southern Iran. Malays J Med Sci. (2015) 22:57–63. 10.1155/2015/357140 28729811 PMC5499771

[96] Poorolajal J Rezaie S Aghighi N. Barriers to medical error reporting. Int J Preventive Med. (2015) 6:97. 10.4103/2008-7802.166680 26605018 PMC4629296

[97] Morrison M Cope V Murray M. The underreporting of medication errors: A retrospective and comparative root cause analysis in an acute mental health unit over a 3-year period. Int J Ment Health Nurs. (2018) 27:1719–28. 10.1111/inm.12475 29762887

[98] Senders J. FMEA and RCA: the mantras; of modern risk management. BMJ Qual Saf. (2004) 13:249–50. 10.1136/qshc.2004.010868 15289625 PMC1743870

[99] Tanaka M Tanaka K Takano T Kato N Watanabe M Miyaoka H. Analysis of risk of medical errors using structural-equation modelling: a 6-month prospective cohort study. BMJ Quality Safety. (2012) 21:784–90. 10.1136/bmjqs-2010-048330 22927491

[100] Vardi A Efrati O Levin I Matok I Rubinstein M Paret G . Prevention of potential errors in resuscitation medications orders by means of a computerised physician order entry in paediatric critical care. Resuscitation. (2007) 73:400–6. 10.1016/j.resuscitation.2006.10.016 17289249

