# Supplementary material for: Interventions to reduce the incidence of medical error and its financial burden in health care systems: A systematic review of systematic reviews
Source: Front Med (Lausanne). 2022 Jul 27;9:875426. doi: 10.3389/fmed.2022.875426 (PMC9363709; doi:10.3389/fmed.2022.875426)
Supplement: Supplementary file 1 [file Data_Sheet_1.docx]

Appendix 1: Search strategy

| Data base | Query |
| --- | --- |
| PubMed | ((((((((((((((((medical errors[MeSH Terms] OR "recording error"[Title/Abstract]) OR "no harm"[Title/Abstract] OR "patient fall*"[Title/Abstract]) OR "hospital infection"[Title/Abstract]) OR "transfusion error"[Title/Abstract]) OR "prescription error"[Title/Abstract]) OR "prescribing error"[Title/Abstract]) OR "CPR error"[Title/Abstract]))) OR "medication error"[Title/Abstract]) OR "near miss"[Title/Abstract]) OR "suicide"[Title/Abstract]) OR "sentinel event"[Title/Abstract]) OR "never event"[Title/Abstract]) AND systematic[sb]) |
| Scopus | ( TITLE-ABS-KEY ( "medical error*" ) OR TITLE-ABS-KEY ( "medical mistake*" ) OR ( TITLE-ABS-KEY ( "recording error*" ) OR TITLE-ABS-KEY ( "no harm" ) OR TITLE-ABS-KEY ( "medication error" ) OR TITLE-ABS-KEY ( "CPR error" ) OR TITLE-ABS-KEY ( "suicide" ) OR TITLE-ABS-KEY ( "patient fall" ) OR TITLE-ABS-KEY ( "hospital infection" ) OR TITLE-ABS-KEY ( "transfusion error" ) OR TITLE-ABS-KEY ( "prescribing error" ) OR TITLE-ABS-KEY ( "prescription error" ) OR TITLE-ABS-KEY ( "near miss" ) OR TITLE-ABS-KEY ( "never event" ) OR TITLE-ABS-KEY ( "surgical error" ) OR TITLE-ABS-KEY ( "sentinel event" ) AND TITLE-ABS-KEY ( "systematic review" ) AND ( LIMIT-TO ( LANGUAGE , "English" )) |
| Embase | 'medical error*':ti,ab,kw OR 'medical mistake*':ti,ab,kw OR 'medication error':ti,ab,kw OR 'CPR error':ti,ab,kw OR 'recording error':ti,ab,kw OR 'patient fall':ti,ab,kw OR 'hospital infection':ti,ab,kw OR 'transfusion error':ti,ab,kw OR 'prescribing error':ti,ab,kw OR 'prescription error':ti,ab,kw OR 'near miss':ti,ab,kw OR 'never event':ti,ab,kw OR 'surgical error':ti,ab,kw OR 'sentinel event':ti,ab,kw OR 'no harm':ti,ab,kw OR suicide':ti,ab,kw AND 'systematic review'/de |

| Ovid Medline | Search |
| --- | --- |
| #1 | medical error*.tw. OR medical mistake*.tw. OR recording error*.tw. no harm.tw. OR medication error.tw. OR CPR error.tw. OR suicide.tw. OR patient fall.tw. OR hospital infection.tw. OR transfusion error.tw. OR prescribing error.tw. OR prescription error.tw. OR near miss.tw. OR never event.tw. OR surgical error.tw. OR sentinel event.tw. |
| #2 | LIMIT: English language |
| #3 | LIMIT: systematic review OR meta-analysis |
