# Supplementary material for: Interventions to reduce the incidence of medical error and its financial burden in health care systems: A systematic review of systematic reviews
Source: Front Med (Lausanne). 2022 Jul 27;9:875426. doi: 10.3389/fmed.2022.875426 (PMC9363709; doi:10.3389/fmed.2022.875426)
Supplement: Supplementary file 2 [file Table_1.docx]

**Supplementary Table 1: Characteristics of included reviews**

| **Overall results** | **Interventions** | **Interventions group** | **Medical error type** | **Number of included studies** | **setting** | **Aim of study** | **Author, year** |  |
| --- | --- | --- | --- | --- | --- | --- | --- | --- |
| Significant 27% reductions in the overall rate of medication errors occurrence | Pharmacist involvement in treatment process | Pharmacists and clinical pharmacists role | Medication Error | 19 | All hospital settings | To qualitatively and quantitatively evaluate the impact of clinical pharmacist interventions on medication error rates for hospitalized pediatric patients | Naseralallah et al., 2020 |  |
| Prescribing errors were reduced. | Pharmacists and computers matching medications  Partnerships with pharmacists  Prescriber education  Medication matching by trained physicians  Computerized physician order entry | Use of electronic systems | Medication Error | 34 | Acute medical and surgical settings | To compare the effectiveness of different interventions in reducing prescribing, dispensing and administration medication errors. | Manias et al., 2020 |  |
| Reduction in prescribing errors | Prescribing Health Information Technology | Use of electronic systems | Medication Error | 35 | All hospital settings | Quantitatively assess the Health Information Technology that reduces prescribing errors in hospitals and identify the behavior change techniques associated with effective interventions. | Devin et al., 2020 |  |
| Led to a decrease of medication errors | Encouraging pharmacist-led education to increase medication error awareness  Incorporating better and innovative pharmacy-related work approaches  Implementing appropriate and secured policies for medication error reporting | Pharmacists and clinical pharmacist role | Medication Error | 7 | All hospital settings | To evaluate pharmacist-centered strategies that has an impact in medication error reduction and prevention. | Gillani et al., 2020 |  |
| Effective in reducing medication errors in all transitions of care | Provision of medication reconciliation in Thailand | Pharmacists and clinical pharmacist role | Medication Error | 7 | All hospital settings | To evaluate the effect of medication reconciliation on the reduction of medication error in Thailand. | Chiewchantanakit et al., 2020 |  |
| Medication error identification and reduction. | Including a pharmacist in discharge planning | Pharmacists and clinical pharmacist role | Medication Error | 38 | All hospital settings | To evaluate programs that provide pharmacy-led continuity of care services and to assess their effectiveness in improving patient outcomes. | Bethishou et al., 2020 |  |
| May be considered to reduce incidence of medical errors and adverse events. | Implementation of safety culture programs | Process interventions | overall medical errors | 16 | Emergency department settings | To narratively summarize the literature reporting on the effect of teamwork and communication training interventions on culture and patient safety in emergency department settings. | Alsabri et al., 2020 |  |
| brief suicide prevention interventions were associated with reduced subsequent suicide attempts (pooled odds ratio, 0.69) | briefcontact interventions, care coordination, safety planning interventions, and other brief therapies | Contact interventions & Process and patient care interventions | Patient suicide | 14 | Acute Care Settings | To examine the association of brief acute care suicide prevention interventions with patients' subsequent suicide attempts | Doupnik et al., 2020 |  |
| Reduction in medication errors and dosing errors | Electronic prescribing strategies | Use of electronic systems | Medication Error | 38 | All hospital settings | To assess the impact of electronic prescribing strategies on medication errors and patient harm in hospitalized patients. | Roumeliotis et al., 2019 |  |
| Interception the highest of error [ Computerized physician order entry :96%], [ computer-assisted prescription:86%] and [ Clinical Pharmacists intervention:80%] | Computerized physician order entry  Computer-assisted prescription  Clinical Pharmacists intervention | Use of electronic systems | Medication Error | 20 | All hospital settings | To analyze clinical studies on medication errors to assess implemented intervention strategies reducing medication errors and measured outcomes. | Shitu et al., 2019 |  |
| Reduction in prescribing errors. | Strategies that are sensitive to local context and designed to increase adherence to insulin prescribing guidelines | Leadership or managerial manners and strategies & Process interventions | Medication Error | 35 | All hospital settings | To identify interventions that are effective in improving insulin prescribing for people with diabetes | Bain et al., 2019 |  |
| Reduction in Prescription errors | Use of clinical decision support tools | Use of electronic systems | Medication Error | 24 | Trauma settings | To evaluate clinically relevant outcomes related to the use of clinical decision support tools for the diagnosis, treatment, and supportive care of patients with cancer. | Pawloski et al., 2019 |  |
| Effective in preventing iatrogenic risk related to medication Error | Simulation | Use of electronic systems | Medication Error | 21 | Oncology wards | To assess whether human simulation in healthcare helps to reduce medication error. | Sarfati et al., 2019 |  |
| Beneficial role of clinical pharmacists in the improvement of quality, safety, and efficiency of patients’ pharmaceutical care in Iran. | Clinical pharmacist interventions regarding designing protocols, improving drug utilization pattern, as well as detection, prevention, and management of medication errors | Pharmacists and clinical pharmacists role | Medication Error | 39 | All hospital settings | To collect data and critically evaluate the clinical and economic effects of Iranian clinical pharmacists interventions and activities. | Noormandi et al., 2019 |  |
| A significant impact on specific outcomes such as patients falls and health care-associated infections | Validation of knowledge through nursing certification | Education and professional skills &  Caregivers’ education and behavioural change interventions | Patients falls and health care-associated infections | 9 | All hospital settings | The purpose was to examine the relationship between nursing certification and clinical outcomes | Coelho, 2019 |  |
| Improvement of medication safety and quality of care, mainly by decreasing medication errors | Drug distribution systems | Process interventions | Medication error | 30 | All hospital settings | To systematically review automated and semi-automated drug distribution systems in hospitals and to evaluate their effectiveness on medication safety, time and costs of medication care | Ahtiainen et al., 2019 |  |
| Reduction in the direct healthcare cost, excessive use of medicines, and prescription errors | Counseling and educating of patients  Working as a member of the healthcare team  Detecting prescribing errors  Reviewing prescriptions on the ward  Giving training and monitoring of the implementation of policies | Use of electronic systems & Leadership or managerial manners and strategies & Process interventions | Medication error | 14 | Primary and secondary settings | To focuse on the impact of clinical pharmacy services in the Nepalese healthcare setup. | Mikrani et al., 2019 |  |
| Reduction in the overall ME rate in the prescription process, as well as specific types of errors, such as wrong dose or strength, wrong drug, frequency, administration route, and drug-drug interaction errors. | computerized prescriber order entry | Use of electronic systems | Medication error | 19 | All hospital settings | To systematically review of published data on the effect of computerized prescriber order entry with clinical decision support on medication error and adverse drug event rates | Velez-Diaz-Pallares et al., 2018 | |
| Pharmacists are better than doctors at adhering to dosing guidelines when prescribing by protocol and make significantly less prescribing errors when charting patients' usual medications on admission to hospital. | Pharmacist prescribing | Pharmacists and clinical pharmacists role | Medication error | 15 | All hospital settings | To synthesize the best available evidence on the safety and effectiveness of pharmacist prescribing on patient outcomes in patients who present to hospital | Poh et al., 2018 |  |
| Decrease in medication errors and an increase in task efficiency | Awareness of interruptions among staff | Leadership or managerial manners and strategies | Medication error | 9 | All hospital settings | To synthesise and summarise data gathered by direct observation of the characteristics of interruptions in the context of nursing medication administration in hospital settings. | Schroers., 2018 |  |
| Impact on nursing workflow and reduction in medication errors | Electronic medication administration records | Use of electronic systems | Medication error | 18 | All hospital settings | To share the results of a preliminary literature review on the impacts of electronic medication administration records on patient safety. | Alanazi et al., 2018 |  |
| Reduction in medical errors | Increased documentation through patient involvement and feedback on the medical file | Patient-centred interventions | Medical error | 12 | All hospital settings | To systematically review the literature on the patient perspective of effects of personal medical record accessibility on the individual patient, patient–physician relationship and quality of medical care. | Vermeir et al., 2017 |  |
| Substantial improvement in patient safety by reducing retained surgical instruments errors | Radio frequency identification technology | Radio frequency identification technology | Surgical errors | 5 | Operating room | To identify the impact of radio frequency identification technology on reducing retained surgical instruments errors and improving patient safety. | Schnock et al., 2017 |  |
| It was associated with an 85% reduction in medication prescribing error rates and a 12% reduction in intensive care units mortality rates. | Transition from paper-based ordering to commercial computerized provider order entry systems in intensive care units | Use of electronic systems | Medication error | 20 | ICU | To conduct a systematic review and meta-analysis of the impact of commercial computerized provider order entry and clinical decision support systems on medication errors, length of stay, and mortality in intensive care units. | Prgomet et al., 2017 |  |
| Significant reductions in postoperative complications and medication-related problems and improved compliance | Use of checklistsusing of checklists. | Process interventions | Healthcare-associated infections& medication error& Surgical error | 9 | All hospital settings | To conduct a systematic review of randomized control trials of checklists to determine their effectiveness in improving patient safety outcomes in hospitalized patients. | Boyd et al., 2017 |  |
| Potential effectiveness in reducing falls and fall rates in acute care hospitals | Patient-centered interventions  Tailored patient education | Educational and professional skills | Patient fall | 5 | Acute care settings | To evaluate the effectiveness of patient-centered interventions on falls in the acute care hospitals. | Avanecean et al., 2017 |  |
| Statistically significant -related improvements in reducing mortality, lengths of stay, medication errors, and hospitalization costs as well as improved staff and patient satisfaction | Inter professional rounding | Process interventions | Medication errors | 10 | All hospital settings | To explore the benefits of Inter professional rounding for patients, clinicians, and the healthcare system | Ashcraft et al., 2017 |  |
| Useful and efficient method in reducing the number of risks and improving service quality | Failure mode and effects analysis technique | Process interventions | Overall medical error | 22 | All hospital settings | To review of risk analysis using Failure mode and effects analysis technique in different hospital wards and departments | Asgari et al., 2017 |  |
| Consistent association with a wide range of patient outcomes such as reduced mortality rates, falls, hospital acquired infections and increased patient satisfaction | Positive organizational and workplace cultures | Organisational and workplace cultures & Managerial and organisational interventions | Patients’ falls & Healthcare-associated infections | 62 | Primary and secondary health settings | To systematically review and synthesize the evidence on the extent to which organizational and workplace cultures are associated with patient outcomes. | Braithwaite et al., 2017 |  |
| Effective strategy in reducing medication error | Pharmacy-led medication reconciliation interventions | Pharmacists and clinical pharmacists role | Medication error | 19 | All hospital settings | To evaluate the impact of pharmacy-led medication reconciliation interventions on medication discrepancies at hospital transitions and to categorize these interventions as single transition interventions or multiple transitions interventions. | Mekonnen et al., 2016 |  |
| Effective on medication errors and adverse drug events reduction and better workflow, and healthcare professional communication | use of hospital electronic prescribing systems | Use of electronic systems | Medication error | 10 | All hospital settings | To summarise the available evidence of the impact of inpatient electronic prescribing on patient safety, with a focus on implications for the UK. | Ahmed et al., 2016 |  |
| Potential reduction in error and costs and improvement in quality within radiology | Lean and Six Sigma Quality Improvement methodologies | Quality improvement methodologies | Diagnostic error | 23 | Radiology department | To systematically assess the literature with regard to the use and efficacy of Lean and Six Sigma within radiology. | Amaratunga et al., 2016 |  |
| Reduction in exposure error and diagnostic errors | Using of computerized physician-order entry  Correct patient identification by 3checks (correct patient, correct site, and correct procedure)  Digital imaging  Double reading | Digital and electronic & Patient identification | Diagnostic errors | 7 | Radiology department | To identify the type and prevalence of errors directly associated with radiography practice and the imaging cycle, with a view to developing recommendations to reduce common errors. | Zhou et al., 2015 |  |
| Significant 43% reduction in preventable adverse drug events and prescribing errors | Pharmacist intervention | Pharmacists and clinical pharmacist role | Medication error | 8 | Intensive care units | To focuses on controlled clinical trials evaluating the effect of pharmacist intervention on medication errors in Intensive care unit settings. | Wang et al., 2015 |  |
| Reduction in costs and medical errors | Use of hospital information systems | Use of electronic systems | Overal medical error | 53 | All hospital settings | To study the impacts of hospital information systems in Iran and the methods used for their evaluation | Ahmadian et al., 2015 |  |
| Reduction but not elimination in programming errors | Use of smart pumps | Smart pumps impact | Medication error | 22 | All hospital settings | To identify the impact of smart pumps on error reduction and on the complex process of medication administration, and strategies to maximize the benefits of smart pumps. | Ohashi et al., 2014 |  |
| Association with a greater than 50% declination in preventable adverse drug events and decreases in medication errors | Implementation of computerized provider order entry adverse drug events | Use of electronic systems | Medication error | 16 | All hospital settings | To assess the effectiveness of adverse drug events at reducing preventable adverse drug events in hospital-related settings, and examine reasons for heterogeneous effects on medication errors. | Nuckols et al., 2014 |  |
| Reduction in patient falls | Hourly rounding programs | Hourly rounding programs | Patient’s fall | 16 | All hospital settings | To synthesize the evidence concerning the effect of hourly rounding programs on patient satisfaction with nursing care and discuss implications for nurse administrators. | Mitchell et al., 2014 |  |
| Potential identification of many medication discrepancies and reduction in potential harm | medication reconciliation and medication review processes | Process interventions | Medication error | 83 | All hospital settings | To examine the evidence regarding the effectiveness of medication reconciliation and review and to improve clinical outcomes in hospitals. | Lehnbom et al., 2014 |  |
| Usefulness reduction in medication administration errors | Automated drug dispensing  Ccomputerized physician order entry Barcode-assisted medication administration with electronic administration records  Nursing education/training using simulation -clinical pharmacist-led training | Use of electronic systems | Medication error | 13 | All hospital settings | To review and critically appraise interventions designed to reduce medication administration errors in the hospital setting. | Keers et al., 2014 |  |
| Reduction in medical errors and adverse drug events , especially when computerized provider order entry systems are bundled with clinical decision support systems designed to alert physicians and other healthcare providers of pending lab or medical errors. | Computerized provider order entry systems | Use of electronic systems | Overall medical error | 50 | All hospital settings | To examine the benefits of and barriers to computerized provider order entry adoption in hospitals to determine the effects on medical errors and adverse drug events and examine cost and savings associated with the implementation of this newly mandated technology. | Charles et al., 2014 |  |
| Reduction in medication errors | Computerized physician order entry with or without clinical decision support systems  Aautomation, computer assisted  Barcode technology  Pharmacists role training  System designs | Use of electronic systems & Pharmacists and clinical pharmacists role | Medication error | 42 | All hospital settings | To review systematically the research literature on the various interventions for providing medication safety in hospitals. | Acheampong et al., 2014 |  |
| Relationships with higher patient satisfaction and lower patient mortality, medication errors, restraint use and hospital-acquired infections | Positive relational leadership styles | Leadership or managerial manners and strategies  & Managerial and organisational interventions | Medication error & Healthcare-associated infections | 20 | All hospital settings | To examine the relationship between and patient outcomes. | Wong et al., 2013 |  |
| Reduction in equipment error | Use of equipment Checklists | Use of checklists and counting materials | Surgical errors | 28 | Operating room | To determine the proportion and characteristics of equipment-related error in the operating room to further improve quality of care. | Weerakkody et al., 2013 |  |
| Lower rate of nosocomial S. aureus infections | Institutionalized prescreening program followed by an appropriate eradication using mupirocin ointment and chlorhexidine soap/shower | Use of medication | Healthcare-associated infections | 15 | Operating room | To critically evaluate the literature and identify modifiable factors to reduce the risk of surgical site infection. | Savage et al., 2013 |  |
| Reduction of clinical error rates for emergency department teams | Inter professional education | Inter-professional education | Overall medical error | 15 | Emergency department | To assess the effectiveness of Inter professional education interventions compared to separate, profession-specific education interventions; and to assess the effectiveness of IPE interventions compared to no education intervention. | Reeves et al., 2013 |  |
| Effectiveness in alerting users about potential clinical hazards and errors during pharmacy order entry | electronic patient medication record systems | Use of electronic systems | Medication error | 5 | Primary and secondary care settings | To systematically explore the literature and synthesize published evidence about the effectiveness of safety features and alerts in electronic patient medication record systems at the point of pharmacy order entry, in primary and secondary care. | Ojeleye et al., 2013 |  |
| Effectiveness in developing countries | Hand-hygiene campaigns  Antibiotic stewardship and other elementary infection control practices | Managerial and organisational interventions & Caregivers’ education and behavioural change interventions | Healthcare-associated infections | 34 | All hospital settings | To evaluate the effectiveness of interventions for preventing nosocomial infections in developing countries. | Murni et al., 2013 |  |
| Reduction in patient falls | Using of screening tools  Staff and patient education  Discreetly displaying high risk of falls  Exercise, safety while ambulating (assistive devices, footwear, etc)  Medication use  Toileting | Education and professional skills & Methods/tools evaluating patients’ fall risk & Process and patient care interventions | Patients’ falls | 15 | All hospital settings | To identify articles for risk stratification and interventions. | Cumbler et al., 2013 |  |
| Reduction in wrong blood in tube | Changes to blood sample labeling  Wweekly feedback  Handwritten transfusion requests  Aan electronic transfusion system  Education  Second check of identification of patients at sampling and confirmatory sampling | Identification of patients (labelling and barcoding) | Transfusion and testing errors | 9 | All hospital settings | To identify interventions that have been implemented and the effectiveness of these interventions to reduce wrong blood in tube incidence in red blood cell transfusion. | Cottrell et al., 2013 |  |
| Efficient in reducing prescription errors | computer-based decision support systems | Use of electronic systems | Medication error | 6 | All hospital settings | To summarize excellent research and to select best papers published in 2012 in the field of computer-based decision support systems in healthcare. | Bouaud et al., 2013 |  |
| Interventions to reduce medication errors included | Publishing monitoring results and linking monitoring results with performance evaluation  Communicating warning information with prescribers  Analyzing the reasons for medical errors  Performing education for warning information of high frequency  Simultaneous technology  Installing prescription automatic screening system | Use of electronic systems & Leadership or managerial manners and strategies | Medication error | 12 | All hospital settings | To systematically assess the outcomes and effectiveness of prescription automatic screening system on rational drug use in China. | Yang et al., 2012 |  |
| Effectiveness for reducing patient specimen and laboratory testing identification errors in diverse hospital settings | Barcoding | Identification of patients (labelling and barcoding) | Transfusion and testing errors | 17 | All hospital settings | To systematically review of the effectiveness of barcoding practices for reducing patient specimen and laboratory testing identification errors | Snyder et al., 2012 |  |
| Minimization in medication error and increase damianimedication safety | A multifaceted, integrated approach involving all aspects of the medication use process, from initial naming of international non-proprietary name through to consumer education | Use of electronic systems & Process interventions | Medication error | 32 | All hospital settings | To develop strategies and recommendations to enhance patient safety and minimize clinical issues with look-alike, sound-alike medication names. | Ostini et al., 2012 |  |
| Economical attractive strategies for improving patient safety and reduction in errors | Pharmacist-led medication reconciliation  The Keystone intensive care unit intervention for central line-associated bloodstream infections  Chlorhexidine for vascular catheter site care  Standard surgical sponge counts | Process interventions & Use of medication & Pharmacists and clinical pharmacists role & Use of checklists and counting materials | Healthcare-associated infections & Medication error & Surgical errors | 5 | All hospital settings | To systematically review comparative economic analyses of patient safety improvements in the acute care setting. | Etchells et al., 2012 |  |
| Statistical significant reduction in the medication error | Double checking | Process interventions | Medication error | 16 | All hospital settings | To evaluate the evidence for double checking the administration of medicines. | Alsulami et al., 2012 |  |
| Reductions in perceived medical errors/adverse events | Computer-enabled discharge communication | Use of electronic systems | Overall medical error | 12 | All hospital settings | To conduct a systematic review evaluating the efficacy of computer-enabled discharge communication compared with traditional communication for patients discharged from acute care hospitals. | Motamedi et al., 2011 |  |
| Significant factors in reducing errors during routine medication rounds | Administration from individually labelled medication and administered close to the patient | Process interventions | Medication error | 19 | Acute care settings | To undertake a systematic review of roles and systems for preventing medication error during routine medication administration in hospital-based acute care settings | Wimpenny et al., 2010 |  |
| Fall the overall rate of medication errors | Error-checking at each stage of the process of preparing and administering intravenous medicines  Removing the reconstitution step by providing prepared injections | Process interventions | Medication error | 9 | All hospital settings | To investigate the overall probability of error in preparing and administering intravenous medicines; to identify at which stage of the process an error is most likely to occur; and to determine the impact of error correction on the error probability. | McDowell et al., 2010 |  |
| Increase physicians' adoption and to reduction in medication errors | computerized physician order entry systems subtle design | Use of electronic systems | Medication error | 19 | All hospital settings | To examine the impact of design aspects of computerized physician order entry systems for medication ordering on usability, physicians' workflow and on medication orders | Khajouei et al., 2010 |  |
| Prevention of suicides | Staffs’ Engagement with patients' family problems  Reduction in absconding without locking the door. | Measures to reduce patients’ absconding and engagement with patient’s family | Patients’ suicide | 98 | All hospital settings | To find what can be learned from the literature about specific circumstances under which suicide seems more likely to occur. | Bowers et al., 2010 |  |
| Most demonstrated benefit in improving prescribing behavior and/or reducing error rates. | Computerized drug alerts and prompts | Use of electronic systems | Medication error | 20 | All hospital settings | To evaluate the efficacy of computerized drug alerts and prompts | Schedlbauer et al., 2009 |  |
| Positive impact | Personal digital assistants  Data management  Rapid response  Error prevention accessibility | Use of electronic systems | Overall medical error & Medication error | 13 | All hospital settings | To examine evidence regarding the impact of mobile handheld technology on hospital physicians' work practices and patient care, focusing on quantification of the espoused virtues of mobile technologies. | Prgomet et al., 2009 |  |
| Preventing medication error  Preventing fall incidents  Decrease of errors | Computerized physician order entry , pharmacists participation in physician education tools, clinical decision support system on a personal digital assistant, bar code technology, an organization-wide safety program, smart-pump technology, structured order sheet, Breakthrough Series evaluation by Silver and Antonow and Failure mode and effects analysis .  Computerized decision support system. human simulation training | Use of electronic systems & Methods/tools evaluating patients’ fall risk & Pharmacists and clinical pharmacists role & Process interventions & Smart pumps impact & Digital and electronic | Medication error & patients’ fall & diagnostic error & overall medical error | 38 | All hospital settings | To synthesize the evidence on the effectiveness of detection, mitigation, and actions to reduce risks in hospitals and to identify and describe components of interventions responsible for effectiveness. | Dückers et al., 2009 |  |
| Similar effects in reducing errors related to a wrong procedure Proactive interventions impacted more positively than reactive ones in reducing medication errors, technical errors and errors due to personnel. | Proactive systematic safety processes and Reactive systematic safety processes | Process interventions | Overall medical error & Medication error | 26 | All hospital settings | To conduct a systematic literature review assessing the impact of systematic safety processes on different error categories. | Damiani et al., 2009 |  |
| Identifying and reducing medication errors | Services provision by pharmacists in the emergency department | Pharmacists and clinical pharmacists role | Medication error | 17 | Emergency department | To ascertain the scope of involvement of clinical pharmacists in the emergency department. | Cohen et al., 2009 |  |
| Considerable reduction in Healthcare-associated infections | Implementation of educational interventions | Caregivers’ education and behavioural change interventions | Healthcare-associated infections | 26 | All hospital settings | To determine the effect of educational strategies of healthcare providers for reducing Healthcare-associated infections. | Safdar et al., 2008 |  |
| Positive effect on medication safety and reduction in medication error rate | Computerized physician order entry | Use of electronic systems | Medication error | 67 | All hospital settings | To identify all published studies evaluating computerized physician order entry in the inpatient setting and uniformly classify these studies on outcome measure and study design. | Eslami et al., 2008 |  |
| Reduction in the risk for medication errors and adverse drug events | Electronic prescribing | Use of electronic systems | Medication error | 25 | All hospital settings | To analyze the relative risk reduction on medication error and adverse drug events by computerized physician order entry systems. | Ammenwerth et al., 2008 |  |
| A large proportion of drug-related nosocomial infections could have been prevented | Avoiding the use of multi-dose vials | Process interventions | Healthcare-associated infections | 128 | All hospital settings | To describe characteristics of Drug-related outbreaks and to determine the most frequent occasions in which contamination of substances for patient care take place. | Vonberg et al., 2007 |  |
| Reduction in Healthcare-associated infections | Behavioral interventions  Formation of a multi-disciplinary quality improvement team  Compliance monitoring and feedback  A mandate to sign a hand hygiene requirement statement | Caregivers’ education and behavioural change interventions & Managerial and organisational interventions | Healthcare-associated infections | 33 | All hospital settings | To evaluate studies testing the effectiveness of interventions aimed at changing healthcare workers' behavior (in reducing Healthcare-associated infections) and to summarize the findings of the studies with the highest quality scores. | Aboelela et al., 2007 |  |
| Risk of acquiring a Nosocomial Infection can be reduced substantially | Regular cleaning of equipment with 70% alcohol | Environment/equipment cleaning | Healthcare-associated infections | 23 | All hospital settings | To determine levels of contamination on healthcare equipment, to identify viable cleaning protocols and to establish the methodological quality of current evidence. | Schabrun et al., 2006 |  |
| Prevention of ventilator-associated and non-ventilator-associated pneumonia | Antibiotic treatment strategies  General preventive strategies  Targeted preventive strategies | Use of medication | Healthcare-associated infections | 18 | All hospital settings | To review the evidence on pathogenesis, diagnosis, treatment, and prevention of both ventilator-associated and non-ventilator-associated pneumonia. | Flanders et al., 2006 |  |
| Increased adherence to guideline-based care  Enhanced surveillance and monitoring  Decreased medication errors | Health information technology | Use of electronic systems | Medication error | 257 | All hospital settings | To systematically review evidence on the effect of health information technology on quality, efficiency, and costs of health care. | Chaudhry et al., 2006 |  |
| Minimization of errors in intravenous drug administration in anesthesia | Systematic countermeasures  Reading the label on any drug ampoule or syringe before a drug is drawn up or injected  Optimization the legibility and contents of labels on ampoules and syringes according to agreed standards  Labelling on syringes  Using of formal organization of drug drawers and workspaces  Double checking of a drug before it is drawn up or administered | Process interventions | Medication error | 98 | Operating rooms | To develop evidence-based recommendations for the minimization of errors in intravenous drug administration in anesthesia. | Jensen et al., 2004 |  |
| Reduction in medication error rates | Use of Computerized physician order entry and isolated clinical decision support systems | Use of electronic systems | Medication error | 12 | All hospital settings | To systematically review the cumulative evidence on the effects of Computerized physician order entry and clinical decision support systems on medication safety. | Kaushal et al., 2003 |  |
